# Role of Mitochondrial Dysfunction in Neuropathy

**DOI:** 10.3390/ijms26073195

**Published:** 2025-03-29

**Authors:** Nidia Espinoza, Vassilios Papadopoulos

**Affiliations:** Department of Pharmacology and Pharmaceutical Sciences, USC Alfred E. Mann School of Pharmacy and Pharmaceutical Sciences, Los Angeles, CA 90089, USA

**Keywords:** diabetes, neuropathic pain, mitochondrial dysfunction, electron transport chain, reactive oxygen species, calcium homeostasis, apoptosis

## Abstract

Diabetes mellitus is characterized by a state of hyperglycemia, which can lead to severe complications if left untreated or poorly managed. Diabetic peripheral neuropathy (DPN) is one common complication. This condition is characterized by damage to the nerves that supply the legs and feet as well as problems with blood vessels, the heart, or urinary tract. To alleviate pain for patients, clinicians resort to long-term treatment regimens of nerve pain medications, which are usually either anticonvulsants or antidepressants. However, little is understood about the underlying mechanisms of DPN. Many pathogenic pathways have been proposed, one of which is mitochondrial dysfunction. Mitochondrial dysfunction includes a range of possible deficiencies given the number of functions controlled by or located in mitochondria, including their core function of bioenergetics. This review focuses on mitochondrial bioenergetics, including respiration/ATP synthesis and reactive oxygen species (ROS) production, as well as calcium homeostasis and apoptosis, and their potential as targets for the effective treatment of diabetic peripheral neuropathy.

## 1. Introduction

As of 2021, 51.6 million people suffer from some form of chronic pain in the US, defined as pain lasting longer than three months [1]. Pain itself can be classified into two categories: nociceptive, resulting from tissue damage, or neuropathic, resulting from damage to nerves [2]. Various conditions and diseases have been identified as leading to chronic pain, but many lack a definitive molecular mechanism, or fault within, that is the root cause of it. One such condition is diabetic peripheral neuropathy (DPN), whose leading cause is poorly managed or undiagnosed diabetes. An area of great interest in chronic pain research is the mitochondria and the multiple processes that take place within these organelles. Deficiencies in these systems have been identified as potential causes of chronic pain [3]. This review will explore whether existing literature supports the hypothesis that mitochondrial dysfunction contributes to the development and progression of diabetic peripheral neuropathic pain. Our search for recent studies utilized databases such as the USC Libraries Database, which encompasses archives, including PubMed. The keywords used were diabetic peripheral neuropathy, mitochondrial dysfunction, chronic pain, reactive oxygen species, electron transport chain, hyperglycemia, calcium, and apoptosis. To achieve our goal, we will first review pain signaling in relation to the nervous system and neuropathic pain in general, then delve into some of the major functions that take place within the mitochondria, followed by studies that investigate if a dysfunction in these functions contributes to DPN. Moreover, we will discuss whether treatment of these defects is enough to alleviate DPN symptoms.

## 2. Neuropathic Pain

**Figure 1:**
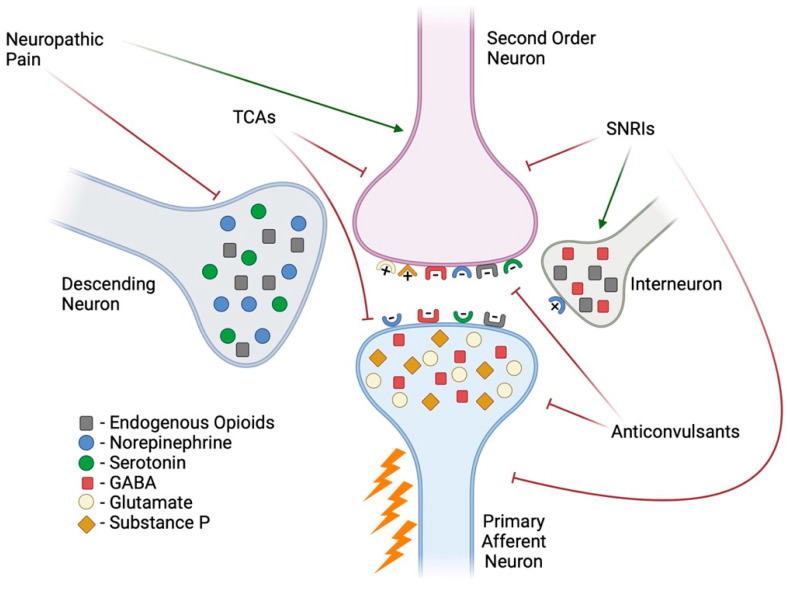
Pain transmission and modulation. A noxious stimulus activates primary afferent neurons that then relay the signal to second-order neurons through the release of excitatory neurotransmitters (e.g., glutamate or substance P). This signal is then sent to the thalamus, where it is processed as pain. In neuropathic pain, there is excessive firing of signals. The endogenous system utilizes descending neurons or interneurons to release inhibitory neurotransmitters (e.g., opiates, GABA, norepinephrine, and serotonin) to stop pain signals from being transmitted [9]. First-line treatments for pain attempt to mimic these endogenous systems. Created in BioRender. Espinoza, N. (2025) https://BioRender.com/v89e665, accessed on 28 January 2025.

Figure 1 depicts the area in which the peripheral and central nervous system meet in relation to pain signal transmission, which takes place in the dorsal horn of the spinal cord, and the signal is then transmitted to the brain. As depicted in Figure 1, the primary afferent neuron is capable of releasing an enormous amount of excitatory neurotransmitters such as substance P and glutamate which then act on their corresponding receptors on the second-order neuron. These neurotransmitters can initiate signaling cascades that result in depolarization of projecting neurons, a larger inward current, and the death of gamma-aminobutyric acid (GABA) interneurons, whose role is to modulate pain through the descending pain pathway, all of which enhance central sensitization [4,5]. Central sensitization is a phenomenon seen in diabetic patients suffering from neuropathy, and it begins with peripheral nerve injury. Activated microglia also contribute to enhanced hyperexcitability within the central nervous system (CNS) through the activation of the p38 mitogen-activated protein kinase (p38MAPK) and the brain-derived neurotrophic factor - tropomyosin-related kinase receptor type B (BDNF-TrkB) signaling pathways, which result in an increase in proinflammatory markers and a reduction in the inhibitory modulation of pain [6,7]. Unsurprisingly, due to its processing and relay role, the thalamus is a CNS region that has been found to be altered in DPN. Studies have shown that neurons in this region fire more spontaneously and at higher rates in DPN [8]. It is important to note that this central sensitization seen in diabetes is not a separate pathology to DPN but a consequence of it that eventually augments pain sensation.

Neuropathic pain is a result of nerve damage that alters the signaling pathways described in Figure 1 and can be defined based on the area affected: autonomic, focal, proximal, or peripheral. In addition, it can be identified based on the cause, such as diabetic neuropathy or chemotherapy-induced neuropathy [10], which are the two most well-studied types of neuropathies. Chemotherapy-induced peripheral neuropathy (CIPN) is estimated to affect 40% of cancer patients and depends on the specific treatment used [11]. For example, the incidence of CIPN in patients treated with oxaliplatin can range from 40–93%, while those who received vincristine have a 20% incidence rate. As time progresses from the last chemotherapy treatment, the incidence of CIPN decreases to 30% at 6 months or more [12]. Longitudinal studies that follow patients for years after chemotherapy treatment are needed to evaluate if the neuropathy subsides; moreover, it is useful for these studies to compare how the duration of chemotherapy treatment influences peripheral neuropathy outcomes of patients [13].

For peripheral neuropathy in general, the first line of treatment falls into three categories: anticonvulsants (i.e., gabapentin or pregabalin), tricyclic antidepressants (i.e., nortriptyline), or serotonin and norepinephrine reuptake inhibitors (i.e., duloxetine) [14]. Due to the complexity of neuropathy and how difficult it is to treat, patients must undergo trial and error with their providers to find the correct medication, frequency, and dosage. Unfortunately, even when patients are successful and find relief, it can be short-lived as they can develop a tolerance to the medications used. Also, some medications come at a cost to their quality of life because the mechanism of action enhances sedation to achieve pain relief through positive modulation of the GABAergic system (Figure 1).

In diabetes, neuropathy is commonly associated with undiagnosed or poorly managed diabetes. Currently, the threshold for a diabetes diagnosis is a glucose concentration of 126 mg/dL or greater on two separate tests [15]. This constant state of hyperglycemia has been associated with the nerve damage that leads to neuropathy [16]. About 50% of diabetic patients end up developing DPN [17], and the most commonly prescribed therapies only provide an estimated 8–13% reduction in pain [18,19].

## 3. Importance of the Mitochondria

The mitochondria, otherwise known as the powerhouse of the cell, have multiple functions that contribute to cell viability and homeostasis [20]. A cell’s main source of energy, adenosine triphosphate (ATP), is synthesized within the mitochondria through the electron transport chain (ETC) via oxidative phosphorylation [21]. Depending on the cell type and energy demand, different types of cells require a larger number of mitochondria. For example, neurons possess a high metabolic rate and, therefore, require a more significant number of mitochondria [22]. The reactions involved in oxidative phosphorylation result in reactive oxygen species (ROS). Although cells can withstand low levels of ROS, processes within mitochondria ensure that levels do not increase to unfavorable amounts, all without compromising the contribution of ROS to signaling pathways. In addition, mitochondria play a crucial role in Ca^2+^ homeostasis and apoptosis, programmed cell death [23].

**Figure 2:**
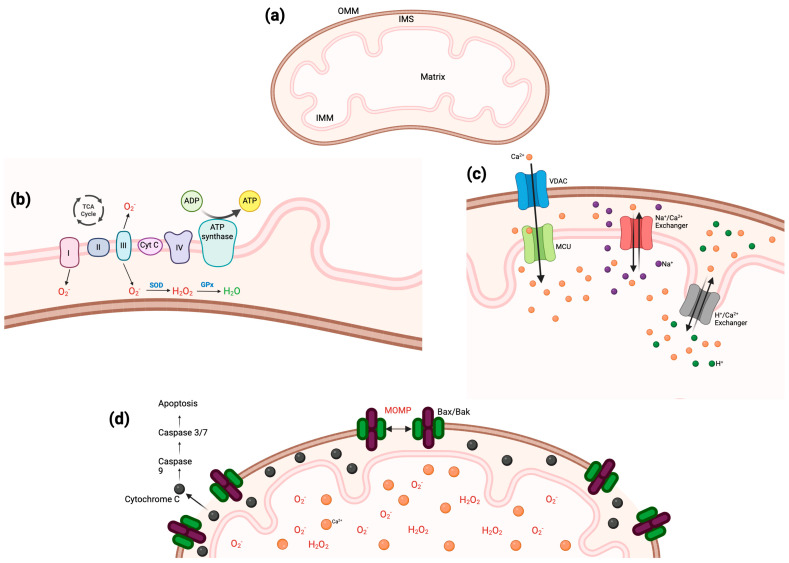
Mitochondrial structure and function. (**a**) Mitochondrial structures. (**b**) ETC complexes and generation of reactive oxygen species. (**c**) Transport of calcium across the OMM and IMM. (**d**) Intrinsic pathways for apoptosis. Created in BioRender. Espinoza, N. (2025) https://BioRender.com/v89e665, accessed on 28 January 2025.

The structure of mitochondria comprises an inner and outer membrane, both working together as well as individually to maintain the health of the organelle. In addition, it includes the intermembrane space, the matrix, and cristae (Figure 2a). The outer mitochondrial membrane (OMM) has direct contact with the cytosol and utilizes porins (e.g., voltage-dependent anion channel, VDAC) that allow ions and small proteins into the intermembrane space. Of particular interest is the translocator protein (TSPO), also located on the OMM. A function of TSPO is to transport cholesterol through the OMM for steroidogenesis, therefore making it the rate-limiting step of this process [24,25]. Along with VDAC1, TSPO is a regulator of mitophagy, and when there is an imbalance in the expression of these OMM proteins, ROS accumulate and lead to mitochondrial dysfunction [26]. The increase in ROS comes from dysregulation of calcium signaling and uptake, which results from phosphorylation of VDAC1, a modification regulated by TSPO [27]. Furthermore, TSPO expression has been shown to be significantly higher in disease states involving neuroinflammation and neurodegeneration [28,29,30]. Various studies have explored the potential for TSPO as a therapeutic target and found promising results. For example, by activating TSPO and the Kv7.2/3 channel simultaneously, researchers were able to promote axon growth in dorsal root ganglion (DRG) neurons in a spine injury model [31]. Moreover, TSPO ligands are also able to significantly decrease oxidative stress and inflammation [32].

The mitochondrial matrix is separated from the intermembrane space by the inner mitochondrial membrane (IMM), which, compared to the OMM, is much less permeable. The IMM is folded throughout the organelle, and these invaginations are known as cristae, which are necessary for mitochondria to preserve their structure. The reduced capacity to cross the IMM requires specific transport proteins to move molecules into the mitochondrial matrix [33].

The mitochondria must be able to respond to various stressors experienced by the cell that require communication with other mitochondria or proliferation or reduction in the number of mitochondria. Fortunately, mitochondria have evolved to perform both fusion and fission to ensure proper response to stressors. For example, the OMM and IMM work in conjunction, through mitofusins and optic atrophy 1, to be able to fuse mitochondria together as needed. In contrast, fission is responsible for dividing the mitochondria and quality control of the number of mitochondria, which again depends on the energy requirement by specific cell types, through endoplasmic reticulum (ER) contact and Dynamin-related protein 1 (Drp1) recruitment [34].

### 3.1. Electron Trasport Chain

The ETC can be found on the IMM cristae and comprises five complexes: (I) NADH dehydrogenase, (II) succinate dehydrogenase, (III) cytochromes b and c1, (IV) cytochrome c oxidase, and (V) ATP synthase (Figure 2b). Mitochondria house numerous biological processes that all work together to maintain cell health. For example, both anabolic and catabolic reactions that take place within mitochondria provide the electrons necessary to fuel the redox reactions of oxidative phosphorylation. These processes include amino acid metabolism and oxidation, nucleotide synthesis, glycolysis, and the TCA cycle. The electrons released from these reactions are carried through complexes I–V by the carriers coenzyme Q and cytochrome c (Cyt c). As the electrons go from one complex to the next, protons are released, thus creating a proton gradient, which is then utilized by ATP synthase to produce ATP [35]. As the energy source of cells, it is critical that mitochondria maintain the ability to continuously and efficiently produce ATP; moreover, it is estimated that each ATP synthase molecule can produce about 100 ATP per second [36]. Of note, in a TSPO knockout cell model, major mitochondrial functions related to the ETC were significantly decreased [37].

A study in 2022 found that mitochondria within neurons have two distinct morphologies: globular or elongated. This study was conducted on mouse cerebellum sections and examined synaptic and axonal mitochondria. The globular mitochondria, a majority of which were found at axonal boutons, exhibited a higher density of cristae and areas of extreme curvature that correlated with more ATP synthase molecules. On the other hand, the elongated mitochondria were both synaptic and axonal mitochondria. The increased number of ATP synthase molecules led the researchers to conclude that globular mitochondria can produce more energy than their elongated counterparts, which correlates with the large energy demand placed on the organelle by neurons [38].

### 3.2. Reactive Oxygen Species

Although many signaling pathways have ROS as a byproduct, the main source of these molecules is the mitochondria. Examples of these molecules are hydrogen peroxide (H_2_O_2_), superoxide (O_2_^−^) (Figure 2b), and hydroxyl radical (^·^OH) [39]; they form part of signaling cascades that direct cell death, growth, and gene expression. ROS also interplay with other mitochondrial functions such as mitophagy and calcium homeostasis. In mitochondria specifically, ROS serve as regulators for mitophagy, which is the process by which damaged mitochondria are degraded [40]. Previous studies have demonstrated that impairments in mitophagy, such as too little ROS, result in an accumulation of defective mitochondria in the cell, leading to various diseases [41]. However, conflicting studies have shown that increased ROS production results in deficient mitophagy pathways, also resulting in pathologies such as DPN [42]. Clearly, there is a fine line between too much and not enough ROS within a cell. It is necessary to elucidate the appropriate levels of ROS that have a positive impact on cell health versus levels leading to various disease states. Nonetheless, ROS are critically necessary for cell signaling and key processes within the nervous system.

Examples of major cell signaling cascades in which ROS play a role are insulin signaling and regulation of transcription factors. The efficient functioning of insulin is crucial for the regulation of glucose levels and the prevention of pathologies such as diabetes, which, unmanaged, leads to multiple complications. When insulin binds to its receptor, it initiates phosphorylation of kinases that ultimately results in the activation of the forkhead box transcription factors (FOXO). However, within this signaling cascade there are various checkpoints that are sensitive to redox regulation, specifically by H_2_O_2_. These include the insulin receptor itself, the RAC-beta serine/threonine-protein kinase (AKT2), FOXO, phosphatase and TENsin homolog (PTEN), and protein tyrosine phosphatase 1B (PTP1B). What makes these molecules susceptible to redox regulation are cysteine residues that, when oxidized, lead to loss of function. Consequences of this regulation include prolonged insulin receptor activation, activation or inhibition of AKT2, in part mediated by PTEN and PTP1B, as well as regulation of genes involved in cell fate, metabolism, or protein homeostasis through FOXO [43]. Transcription factors are similarly controlled by ROS. Studies have demonstrated that ROS can regulate nuclear factor erythroid 2-related factor (NRF2), nuclear factor kappa B (NF-κB), and hypoxia-inducible factor 1α (HIF1α) [44,45,46]. These transcription factors are involved in processes such as stress response, inflammation, and energy promotion through glycolysis, all of which are essential for cell viability.

ROS support mechanisms involving synaptic plasticity, neuronal growth, and function. Under normal conditions, when ROS are present at low levels, they serve as secondary messengers for neurotransmission and differentiation. Moreover, these molecules are essential to neurons throughout their entire lifespan, from neurogenesis to apoptosis. In the developing neuron, ROS inhibit phosphatases, such as PTEN, and allow for kinases, such as PI3, to mediate cellular polarization [47,48]. Recently, it was shown in adult hippocampal progenitor cells that H_2_O_2_ can regulate their growth, and if ROS production is inhibited, neurogenesis comes to a halt [49,50]. For the nervous system to transmit signals well, a neuron’s axon must have the correct growth and polarization. When ROS are present, axons and dendrites form and mature appropriately; however, when H_2_O_2_ production is inhibited, axon length is significantly shorter [51]. Moreover, learning and memory are also greatly impacted by ROS. When hippocampal slices were subjected to N-methyl-D-aspartate (NMDA) receptor activation, an accumulation of superoxide followed. This ROS molecule was shown to modulate extracellular signal-regulated kinase (ERK) and protein kinase C (PKC), both of which are needed for long-term potentiation, a form of synaptic plasticity [52]. Although ROS have always been categorized as injurious to cells, more evidence is emerging that their effects are dependent on their location, cell type, and concentration.

### 3.3. Calcium Homeostasis

Calcium has been extensively studied as a second messenger involved in many processes ranging from cell migration to neuron excitability and even mitochondrial dynamics. It is mainly stored within mitochondria and the ER. It is crucial that calcium levels remain under control for various signaling cascades and cellular communications to work properly. Mitochondria serve as calcium level regulators through two methods: (1) buffering capacity and (2) interactions with other cell components. The concentration of calcium that mitochondria can buffer varies from 50 to 500 nM, depending on the cell type [53]. Therefore, when a cell is exposed to temporary levels of excessive calcium, the mitochondria can actively compensate and allow the cell to return to its normal state.

Calcium transport mechanisms through the mitochondria are determined by what membrane it needs to pass. For transversing the OMM, Ca^2+^ makes its way through the VDAC under conditions of high membrane potential. Calcium can also regulate the transport of other ions and molecules through the VDAC [54]. As for the IMM, Ca^2+^ can permeate in two ways: (1) through an electrophoretic uniporter or (2) exchanged for other cations, contingent on the tissue. The mitochondrial calcium uniporter (MCU) on the IMM uses negative potential to facilitate the transport of calcium. Calcium concentration must reach 1mM or higher for the MCU complex, mitochondrial calcium uptake 1 (MICU1), mitochondrial calcium uptake 2 (MICU2), and the essential MCU regulator (EMRE) to open and allow the passage of calcium ions. In brain and muscle tissues, calcium utilizes a Na^+^/Ca^2+^ exchanger, but a H^+^/Ca^2+^ one can be found in other tissues (Figure 2c). For cells such as neurons, the mitochondria must fine-tune the exchange of various cations, including Na^+^, Li^+^, K^+^, and H^+^, to maintain the outflow of Ca^2+^ from the mitochondrial matrix [55].

The ER is the second key source of calcium for the cell. The mitochondria and ER communicate through Ca^2+^ signaling, and this regulates mitochondrial function. This cross-talk is facilitated by mitochondria-associated membrane (MAM) domains, which entail multiple proteins and receptors. Within the MAM, the ER and mitochondria are tethered together through the vesicle-associated membrane protein-associated protein B (VAPB) and the protein tyrosine kinase phosphatase interacting protein 51 (PTPIP51). In addition, mitofusins (MFN1, MFN2) are also active in MAMs to aid in connecting the ER and mitochondria. The ER utilizes IP3 receptors to send calcium directly into the mitochondria through the VDAC [56]. Interestingly, the mitochondrial TSPO has also been found to be present in MAMs, binding to the VDAC and involved in calcium homeostasis [27,57,58]. This increase in mitochondrial calcium contributes to the downstream effects of Ca^2+^ on mitochondrial function, such as increased ATP synthesis, activation of mitochondrial dehydrogenases, and adenylate transport [59]. Mitochondrial dynamics such as fission, motility, and mitophagy are also regulated by Ca^2+^. This critical cation can induce phosphorylation of Drp1, arrest or mobilize the organelle to where buffering or ATP synthesis is needed, and initiate the PTEN-induced kinase 1 (PINK1) mitophagy pathway [60,61,62]. Specifically for diabetes, disrupted or inefficient calcium signaling between the ER and mitochondria of pancreatic beta cells has been linked to an increased risk of developing this condition [63,64].

### 3.4. Apoptosis

Programmed cell death, otherwise known as apoptosis, is the mechanism by which damaged cells are designated for disposal to maintain physiological homeostasis. In mitochondria, the OMM plays a critical role in regulating signaling cascades that activate apoptosis, through the activation of caspases. This is known as intrinsic apoptosis, and it occurs when the permeability of the OMM is compromised or there is irreversible mitochondrial outer membrane permeabilization (MOMP) [65]. Previous studies have identified how MOMP comes about, and it involves the accumulation of ROS under stress conditions and sustained opening of the mitochondrial permeability transition pore (mPTP) with Ca^2+^ overload [66]. Under normal conditions, the OMM contains antiapoptotic proteins that help prevent MOMP, but when there are cytotoxic stimuli or stressors present (e.g., increased ROS), proapoptotic proteins are able to increase the concentrations of Bax/Bak at the OMM, inducing MOMP. This creates pores in the OMM, which allows for Cyt c to escape the intermembrane space into the cytosol and initiate apoptosis through activation of caspases 9, 3, and 7 (Figure 2d) [67]. Similarly, when there are high levels of Ca^2+^ (overload) and the mitochondrial permeability transition pore (mPTP) is sustained, it leads to swelling and eventual rupture of the OMM, which also releases Cyt c and activates caspases [68]. Even in the absence of caspases, MOMP results in apoptosis through the translocation of endonuclease G, an apoptosis-inducing factor, to the nucleus [69].

The mitochondria are appropriately dubbed the powerhouses of the cell, in more ways than just ATP production. Moreover, any dysfunction in the multiple processes they contribute to and control can have a detrimental effect on health. In the next section, we will review how mitochondrial dysfunction contributes to neuropathy and potential targets to treat such a debilitating condition.

## 4. Mitochondrial Dysfunction and DPN

The pathology of pain is challenging to diagnose and treat, as clinicians rely on patient-reported symptoms and many patients develop tolerance to medication. Treatment regimens are dependent on the type of pain, nociceptive or neuropathic, as well as the pathological cause of the pain. Our review focuses on DPN, and as mentioned above, the common treatments are, for the majority of patients, ineffective at treating their symptoms of neuropathy. Moreover, these treatments are also not very successful at treating other forms of neuropathy (e.g., idiopathic sensory polyneuropathy and CIPN) [70,71]. The description of symptoms varies from patient to patient, so the description of pain intensity can be dull, throbbing, aching, burning, or sharp. In addition, some patients report a feeling of pins and needles while others report numbness and loss of feeling. Lastly, it is important to note that patients who suffer from neuropathy convey both hyperalgesia as well as allodynia, the latter being when innocuous stimuli elicit a painful response versus the former being a noxious stimulus causing a heightened painful sensation [72]. In the following sections, we will review previous studies that examine the mitochondrial functions discussed above and their contribution to DPN. Furthermore, the consequences of mitochondria dysfunction and their current status in clinical trials are summarized in Table 1.

### 4.1. ETC and DPN

The importance of proper mitochondrial respiration and ATP synthesis has been extensively studied. The impact of hyperglycemic conditions (i.e., diabetes) on these cellular mechanisms is of particular interest due to the potential contribution to the development of DPN. Neuropathy itself is defined as pain resulting from nerve damage, and considering the reported symptoms of diabetic patients, this metabolic disease leads primarily to peripheral neuropathy. It is believed that, in DPN, it is the sensory nerves stemming from the dorsal root ganglion (DRG) that supply the hands and feet that are the most damaged with diabetes [73].

One of the most commonly used methods to study DPN is streptozotocin (STZ)-induced diabetes, whose protocol can vary depending on the species and strain of the animal model [74]. A 2010 study utilizing this method found that diabetes decreased mitochondrial respiration of complex I by 31–44% and the capacity of complex IV by 29–39% after just 22 weeks. More importantly, it found that this decreased mitochondrial respiration was reversed with insulin treatment, but no other diabetic symptom was resolved [75]. A more recent study of both in vivo and in vitro models of DPN found similar results of diminished mitochondrial respiration chain complex activities. These STZ animals demonstrated hypersensitivity in thermal and mechanical behavioral tests, reduced nerve conduction, and decreased expression of the Nrf2 transcription factor. In this study, bardoxolone methyl treatment was able to reverse hyperalgesia, improve sensory nerve conduction, and prevent any damage to mitochondrial oxygen consumption. Although this study utilized a drug that failed Phase 3 clinical trials due to severe adverse events, the results are promising and can provide a path to follow in search of more effective treatments for DPN patients than those currently available, specifically pursuing the Keap1-Nrf2-ARE pathway targeted by bardozolone methyl [76].

Another potential pathway that is involved in the reduced maximum oxygen consumption and respiration capacity is NF-κB. In regard to neurons, NF-κB expression and activity is significantly reduced in animal models of DPN. Furthermore, ciliary neurotrophic factor is able to stimulate axon regeneration in sensory neurons and studies have also demonstrated reduced activity in STZ models. With only 24 h of exposure to ciliary neurotrophic factor, DRGs of STZ-treated mice showed a reversal of the deleterious effects on mitochondrial bioenergetics through NF-κB activation [77,78,79]. Lastly, another transcription factor that can undo the effect of hyperglycemia on mitochondria and neurons is the CCAAT/enhancer-binding protein (CEBPβ). This transcription factor regulates insulin-like growth factor 1 (IGF-1), which has been shown to be reduced in diabetes, along with a decrease in the transcription factor itself [80]. Due to the multitude of effects that diabetes can have at the molecular level, it is critical for regulators like transcription factors to respond to cellular stressors and prevent any damage before peripheral neuropathy develops.

### 4.2. ROS and DPN

ROS commonly have a negative connotation; however, previous studies have illustrated their contribution as regulators of critical signaling cascades. In a normal state, low levels of ROS are usable byproducts of oxidative phosphorylation, but in a state of deficient or dysfunctional mitochondria, elevated levels of ROS are present, and this results in oxidative damage. Moreover, previous studies have demonstrated that both hyperglycemia and increased ROS levels, 10 μM of H_2_O_2_, inhibited neuronal differentiation [81]. For DPN, high levels result in the degeneration of neurons, contributing to the development of DPN. As ROS are free radicals, it would seem plausible to hypothesize that treatment with antioxidants would neutralize them and prevent or reverse any damage done, but this is not always the case. Various antioxidants have been studied in both animals and humans, such as alpha lipoic acid (ALA), vitamins E and C, and melatonin, with only ALA receiving approval for the treatment of DPN in Germany. Unfortunately, no set guidelines or target levels have been established for antioxidant enzyme activity or ROS levels. Most studies focus on investigating whether the antioxidant treatment can reduce ROS levels while simultaneously alleviating DPN symptoms [82]. On one hand, antioxidants such as quercetin have been proposed to successfully prevent and treat DPN. In an STZ animal model, the ability to reverse mechanical hypersensitivity and reduce ROS production levels was exhibited [83]. On the other hand, antioxidants such as diosgenin or Dunaliella salina powder worsened neuropathy symptoms [84,85]. A 2025 study explored a novel delivery system, gold nanodots, to deliver vascular endothelial growth factor and assess its effectiveness in treating DPN. The researchers successfully illustrated the efficacy of this treatment to scavenge ROS, increase nerve conduction velocity, restore a normal pain threshold for heat, and enhance the structure of the sciatic nerve, the most common patient-reported nerve as the focal point of their DPN [86].

Previous studies have also identified specific posttranslational modifications that play a role in the development of DPN, such as phosphorylation, acetylation, and SUMOylation. The latter was shown to have neuroprotective properties, and when it is lacking, the cell accumulates elevated levels of ROS and damage to neurons increased [87]. Interestingly, one of the pathways previously mentioned as a potential target as treatment for DPN, the Nrf2 signaling cascade, serves as a major regulator of antioxidant response elements [88]. These include glutathione peroxidase, superoxide dismutase, and thioredoxin, which can undergo glycosylation under hyperglycemic conditions, thus resulting in deficient antioxidant properties [89]. There is considerable literature that highlights both the pros and cons of ROS. Due to the sensitivity of cells to ROS and small differences between necessary ROS levels and levels that cause nerve damage, it may prove difficult for this to be a successful therapeutic target. Nonetheless, examining the effect of other potential treatments for DPN via ROS is crucial as their contribution to the development and progression of DPN is well established.

### 4.3. Calcium and DPN

One of the main drivers of neuropathy is the spontaneous, uncontrolled firing of peripheral neurons, which transmit pain signals and lead to a chronic pain state. In the ascending and descending pain pathways, there are checkpoints in which the endogenous system can regulate a pain signal, but this process is altered under pathological states. For a pain signal to be transmitted, excitatory neurotransmitters such as glutamate and substance P must be released into the synapse. Modulation of this pain signal is performed through the release of molecules such as serotonin, norepinephrine, opiates, and GABA. To mimic this, the first-line treatments for neuropathy are all positive modulators that are meant to increase the amount of these inhibitory neurotransmitters [90]. Moreover, an important regulator for the release of neurotransmitters, regardless of their effect on pain, is calcium [91]. So, if calcium homeostasis within a neuron is not maintained, it can lead to spontaneous firing or inadequate modulation of pain signals, resulting in neuropathy.

Calcium is a key second messenger in many cell signaling cascades; therefore, it is not surprising that an imbalance can cause such a detrimental effect on health. Calcium is able to cross membranes through various types of channels, and these channels and the tissue in which they are located will determine the resulting current. The thalamus is the main tissue through which a pain signal is relayed and processed. Thalamic neurons utilize a T-type current to fire action potentials, which is mediated by the Cav3 subfamily of voltage-gated calcium channels [92]. Animal models of DPN have illustrated that diabetes causes a significant increase in the density of T-type calcium currents while simultaneously lowering the threshold for neuronal firing. Furthermore, the same group of researchers successfully showed that, through gene therapy, silencing Cav3.2 significantly alleviated the hyperalgesia seen in the STZ-induced model of neuropathy [93]. Unfortunately, a 2015 preclinical trial also targeting this channel found that blocking the channel was insufficient at providing pain relief to adult DPN patients [94]. Even though these promising animal study results were not translatable to humans, T-type calcium channels may still prove to be a potential therapeutic target using a different approach.

Multiple processes in which calcium plays a role have been proposed as the cause of DPN, including calcium dynamics in mitochondria. DPN is characterized by significantly increased levels of calcium in both mitochondria and neurodegeneration, so one study explored the effect of preventing calcium influx. They found that, by eliminating the MCU, neurodegeneration was prevented, and the hyperalgesia and allodynia caused by diabetes was reversed [95]. These results suggest that targeting calcium entry into mitochondria can be effective at providing patients with relief. However, due to calcium’s integral function in a multitude of other processes, it remains to be seen if a highly selective treatment for damaged neurons in DPN is possible.

**Table 1:** Effect of mitochondrial dysfunction. Each facet of deficient mitochondria has unique consequences. However, all share the result of increased pain and hypersensitivity in a DPN model. Most studies have demonstrated the possibility of ameliorating negative effects, but no major strides have been made in translating them into potential therapeutics for patients.

Mitochondrial Dysfunction	Consequence	Reversible?	Available Treatment?
Deficient ETC	Increased hypersensitivity, reduced ETC complex activity and protein levels, high levels of ROS and inflammation [96,97]	Yes, to some extent	None
ROS Overload	Sciatic nerve lesions, increased pain sensitivity, deformations in sciatic nerve, slower motor and sensory nerve conduction, decreased blood flow to sciatic nerve, increased apoptosis rate [86,98,99]	Yes, to some extent	None Studies evaluating the effect of potential antioxidants are under way [100]
Disrupted Calcium Homeostasis	Lower threshold for neuron excitation, increased apoptosis rate, Schwann cell demyelination, motor deficits, hypersensitivity, increased T-type channel density [101,102,103]	Yes, to some extent	None Calcium infusions were studied, but no promising results [104] Vitamin D also studied, but no results shared [105]
Dysregulated Apoptosis	Reduced activity of NGF/Akt, GSK3β signaling pathway, increased levels of BAX, decreased BCL2 levels, axonal shrinkage, neuron degeneration, increased hypersensitivity [106,107]	Yes, to some extent	None

### 4.4. Apoptosis and DPN

Excessive use of any signaling pathway will ultimately lead to unwanted effects. However, the role of apoptosis in DPN is more straightforward than other contributing factors or pathways. The pathology for DPN entails a significantly higher degree of apoptosis in neuronal cells, leading to neurodegeneration and aberrant neuron firing. Most of the literature focuses on elucidating the link between apoptosis and DPN to determine if a molecule or treatment is able to inhibit apoptosis and alleviate the effects of DPN [108,109,110]. The important distinction to consider in reviewing these studies is whether the molecule was successful and if it inhibited caspase-dependent or caspase-independent apoptosis; the latter is not always evident. For example, bone morphogenetic protein 5 successfully reversed the symptoms and indices seen in STZ-induced DPN by downregulating cleaved caspases 3 and 9 via phosphorylation of Smad1/5/9 [111]. Similar inhibition of caspase-dependent apoptosis has been reported in other studies utilizing drastically different treatments such as nanoparticles, antioxidants, and knockdown of chemokine ligands [112,113,114]. Regardless of the treatment, the commonality between studies suggests that the importance of apoptosis as a therapeutic target in the development and progression of DPN is to inhibit the process. Many different pathways can activate apoptosis as a downstream effect; however, in mitochondrial dysfunction, the pathways of interest would be those involved in keeping the permeability of the OMM intact while inhibiting apoptosis. It is important to note that apoptosis is not the only method of cell death that has been studied in relation to DPN. Other forms include pyroptosis, necroptosis, and ferroptosis. Moreover, similar to apoptosis, studies have shown that the signaling cascades involved in these forms of cell death are activated by an accumulation of ROS [115,116,117].

## 5. Conclusions

Chronic pain is a highly complex condition, having various causes at the molecular level. Here, we discuss neuropathic pain in relation to mitochondria. Unfortunately, there are no current treatments that target mitochondrial function with an indication for DPN. However, several studies have evaluated the potential of the mitochondria as a therapeutic target for neuropathy. One study investigating whether antioxidants intended to treat the mitochondria specifically could alleviate pain caused by sciatic nerve injury concluded that mito-TEMPO, a nitroxide conjugated with a triphenylphosphonium moiety, was successful at relieving neuropathic pain due to its ability to increase the withdrawal threshold and protect the mitochondria [118]. In addition, the authors reported that the treatment significantly decreased the expression of Drp1, whose overexpression was previously shown to increase pain sensitivity. Moreover, in neuropathic pain, Drp1 has been linked to imbalances in ROS homeostasis, the formation of mPTP through Bax/and Bak, as well as regulation by calcium signaling pathways, all of which support our hypothesis that mitochondrial dysfunction contributes to the development and progression of DPN [119] and thus could serve as a therapeutic target. Mitochondria are complex organelles that house processes essential to maintaining cell homeostasis and ensuring cell health. Under the pathological condition of diabetes, mitochondria become compromised and are unable to function properly. This negatively alters their ability to respond to external stressors and distorts the mechanisms that maintain cell viability. In neurons, these distortions result in neurodegeneration, spontaneous neuron firing, and, ultimately, DPN. Moreover, mitochondrial dysfunction is a broad term used to describe any deviation from normal functionality, such as (1) decreased mitochondrial respiration through the ETC, (2) increased levels of ROS production, (3) imbalances in calcium levels, and (4) increased rates of apoptosis. It is imperative to remember that all these processes are interconnected within the mitochondria. Both DPN and mitochondrial dysfunction are multifaceted concepts, and a straightforward cause and effect link between the two cannot be made yet. However, further investigation into how mitochondrial dysfunction contributes to the development and progression of DPN is necessary to expand on our current understanding and hopefully identify more successful therapeutic targets.
